# Protein Phosphatase 2A (PP2A) Regulates EG5 to Control Mitotic Progression

**DOI:** 10.1038/s41598-017-01915-w

**Published:** 2017-05-09

**Authors:** Yang Liu, Zhong Zhang, Hui Liang, Xuyang Zhao, Ling Liang, Guangxi Wang, Jingyi Yang, Yan Jin, Michael A. McNutt, Yuxin Yin

**Affiliations:** 1 Department of Pathology, Institute of Systems Biomedicine, School of Basic Medical Sciences, Beijing Key Laboratory of Systems Biology, Peking University Health Science Center, 38 Xueyuan Road, Beijing 100191, P.R. China; 2 Peking–Tsinghua Center for Life Sciences, Peking University, Beijing 100871, P.R. China

## Abstract

EG5 (KIF11) is a member of the kinesin-like protein family involved in centrosome separation and bipolar spindle formation. When a cell enters mitosis, CDK1 phosphorylates EG5 at Thr926 and promotes EG5 localization on the mitotic spindle which drives bipolar spindle formation. EG5 provides power for spindle movement and thus controls the dynamics of spindle assembly. However, little is known about EG5 regulation or how EG5 detaches from the spindle upon mitotic exit. In this study we identify EG5 as a novel substrate of PP2A phosphatase, and we show that the PP2A/B55α complex plays an important role in mitotic exit by a mechanism involving EG5. The PP2A/B55α complex physically associates with the EG5 C-terminal tail domain and dephosphorylates EG5 at Thr926 that enables mitotic exit. Conversely PP2A knockdown cells show a high level of phospho-EG5 in late metaphase, which is associated with a delay in mitotic exit. These phenotypic features are similar to those induced by EG5/T926D transfection that mimics phosphorylated EG5 status. Our results argue that PP2A controls mitotic exit through EG5 dephosphorylation. Lack of PP2A leads to abnormal EG5 activation, resulting in delay of mitotic exit.

## Introduction

Protein kinases and phosphatases generate opposing forces in mitotic regulation, and play important regulatory roles in mediating the dramatic changes in mitotic cells such as nuclear breakdown, spindle assembly and cell division^1, 2^. When entering mitosis, a variety of mitotic proteins are activated by mitotic kinase phosphorylation, including CDK1/cyclinB, AuroraB, and PLK1 which trigger and direct this abrupt morphologic transition^3–7^. In contrast, during the interval of mitotic exit, most mitotic phosphorylation is abolished by dephosphorylation and proteolysis by phosphatases such as PP1 and PP2A^1, 8–11^. Failure of timely removal of these phosphorylation signals may lead to high mitotic protein activity and result in an accumulation of errors, or a delay or failure in mitotic exit^12, 13^.

Protein phosphatase 2A (PP2A) is a multifunction phosphatase that is ubiquitously expressed in eukaryotic cells which consists of a complex with three subunits including a scaffold subunit A, a catalytic subunit C, and a regulatory subunit B^14, 15^. The four families of B subunits (B, B′, B″, B′″) specify the PP2A substrate. Through pairing with various regulatory B subunits, PP2A is involved in a variety of cellular functions, including cellular growth, transformation, DNA replication, mitosis, and apoptosis^16–19^. Inhibition of PP2A dephosphorylation significantly delays exit from mitosis, reflecting the importance of PP2A in the regulation of mitotic exit^11, 20^. CDK1 is a key regulator of the mammalian cell cycle^21^, and its degradation follows activation of the anaphase-promoting complex (APC). PP2A has been reported to control mitotic exit by inactivating CDK1 and CDC25^18^. However, absence of CDK1 activity alone is insufficient to induce mitotic exit if PP2A is suppressed by okadaic acid^22^, suggesting other PP2A protein substrates are also required for mitotic exit.

The metaphase-anaphase transition is a complex series of events which mark the beginning of mitotic exit. Sister kinetochores attach to opposite poles of the spindle and align at the metaphase plate, and APC promotes mitotic exit^23, 24^. Sister chromatids separate and move to opposite poles following the spindle fibers^25^. This progressive movement of the spindle and chromosomes is a prominent morphologically identifiable change driven by motor proteins such as dynein and kinesins that generate the forces which result in chromosome separation.

EG5 is a plus-end motor protein which is a member of the kinesin superfamily that plays a critical role in the maintenance and assembly of the bipolar spindle during mitosis^26^. At the onset of mitosis, CDK1 phosphorylates EG5 at Thr926, which promotes localization of EG5 in the form of a homotetramer on the spindle, with heads attached to antiparallel microtubules^27^. The motor domain at the head of EG5 hydrolyzes ATP to generate energy for the movement of EG5 along the microtubule, creating an outward force for spindle separation. However, it is the EG5 N-terminal motor domain which contains the microtubule binding regions. There is substantial evidence that phosphorylation of Thr926 in the EG5 C-terminal region by CDK1 is essential for EG5 activation and localization to microtubules^27–29^. Failure of EG5 attachment to the spindle at mitotic entry may disrupt spindle pole separation, resulting in a monopolar spindle which can lead to catastrophic failure in chromosome segregation and ultimately to cell death^26^. However, the mechanism and timing of EG5 inactivation and EG5 detachment from the spindle at mitotic exit, and the consequences of failure of EG5 detachment are unknown.

Recent studies have suggested that the PP2A/B55α complex acts as a key factor in mitotic spindle breakdown and mitotic exit^30^. Depletion of PP2A/B55α in mammalian cells prolongs mitotic exit, but the mechanism by which PP2A participates in mitotic exit regulation is unclear. In this study, we identify EG5 as a novel PP2A substrate, and show PP2A functions in regulation of mitotic exit. We also evaluate the timing and role of PP2A dephosphorylation of EG5 in mitosis and the consequences of failure of dephosphorylation.

## Results

### PP2A knockdown leads to metaphase delay in HeLa cells

**Figure 1:**
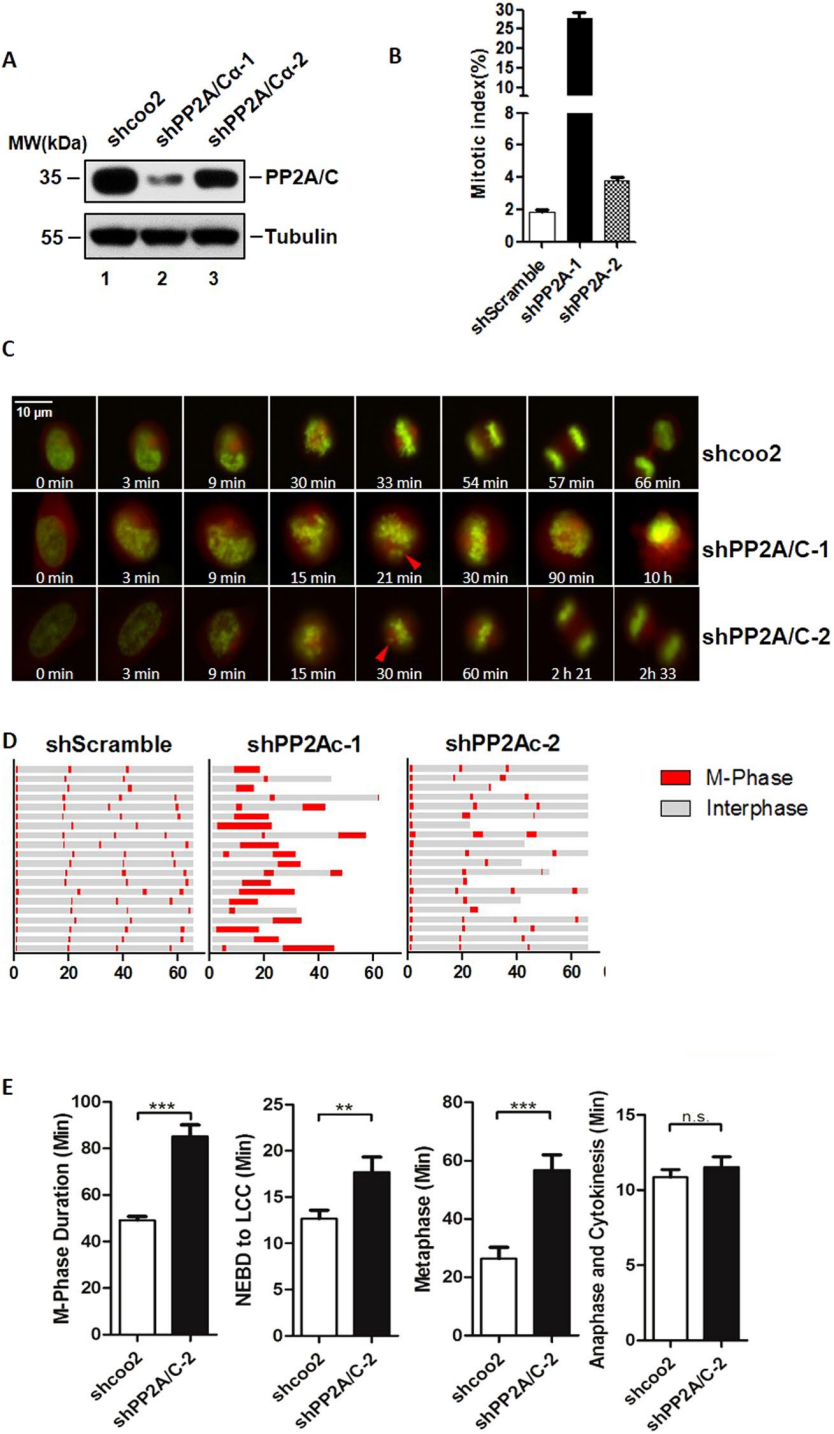
PP2A knockdown leads to metaphase delay in HeLa cells. (**A**) Protein depletion levels of PP2A/C evaluated by Western blot in cells after 48 hours infection with indicated shRNAs. For the original uncropped western blot data, see Supplementary information. (**B**) Mitotic index (MI) of cells (**A**), was calculated by counting metaphase cells in approximately 1000 cells. (**C**) Snapshots of Hela cells (shcoo2, shPP2A/C-1, shPP2A/C-2) taken at indicated times (scale bar indicates 10 μm). Red triangles indicate misalignment which has occurred during metaphase. (**D**) Cell cycle progression of HeLa cells infected with lenti-viruses (shcoo2, shPP2A/Cα-1, and shPP2A/Cα-2). Cells were monitored by time-lapse fluorescence microscopy. H2B-GFP and DsRed-α-tubulin were stably expressed in HeLa cells to indicate chromatin status. Single cells were monitored for 65 hours. Gray sections on the progress bar represent interphase, and red sections represent mitotic phase. (**E**) Mean length of M-phase, NEBD-LCC (Nuclear envelope breakdown to last chromosome congress), Metaphase and anaphase in HeLa cells infected with shPP2A/C-2 and shcoo2 lenti-virus (N > 50).

PP2A is involved in many mitotic processes and PP2A knockdown has been reported to trigger cell death in a variety of cell types^31^. As the extensive cell death resulting from PP2A knockdown may obscure the role of PP2A in mitotic progression, we generated HeLa (human cervical carcinoma) cells containing 2 different shRNAs targeting PP2A/Cα. Both of these shPP2As attenuated expression of PP2A as evaluated by Western blot, however shPP2A/Cα-1 knock down efficiency was greater than that of shPP2A/Cα-2 (Fig. 1A). To evaluate the effect of PP2A knock down, over 1000 cells were imaged and the mitotic index was calculated as the percentage of cells undergoing mitosis. The mitotic index (MI) of shPP2A/Cα-1 (26%) was dramatically higher than the MI of control cells (2%), while shPP2A/Cα-2 increased the mitotic index to just 4% (Fig. 1B). To examine cellular events following PP2A knockdown, live cell imaging was carried out over 65 hours with these two knock down cell lines and a control cell line, and mitotic progression was observed in over 60 representative cells from each cell line. We found HeLa cells with shPP2A/Cα-1 did not align at the metaphase plate, failed to complete mitosis, and underwent cell death (Fig. 1C, middle row, Fig. 1D), which is consistent with what has previously been reported^31^. PP2A/Cα knock down HeLa cells generated with shPP2A/Cα-2 which causes less attenuation of PP2A/C expression than shPP2A/Cα-1 completed mitosis, but required more time for division of sister chromatids than the control cells (Fig. 1C, bottom row, Fig. 1E). The difference in the extent to which mitosis was retarded or stalled in these two shRNA knock down lines implies that any loss of PP2A is detrimental to mitotic progression.

The mean duration of the entire M-phase increased from 49 minutes to 85 minutes after PP2A knockdown with shPP2A/Cα-2, while the mean duration of metaphase increased from 26 minutes to 56 minutes (Fig. 1E). Metaphase was markedly delayed in cells treated with shPP2A/Cα-2, and this delay was proportionately longer than the delay in prometaphase or anaphase. This suggested that the specific quantity of PP2A is crucial for the timing of metaphase-anaphase transition. On the other hand, based on correlation of the strength of shRNA knockdown with the severity of the observed outcomes, we concluded that unlike severe depletion of PP2A/C which abolishes the ability of the cell to complete mitosis, inadequate levels of PP2A/C simply delay the completion of mitosis at metaphase.

### EG5 is a novel PP2A-binding protein

**Figure 2:**
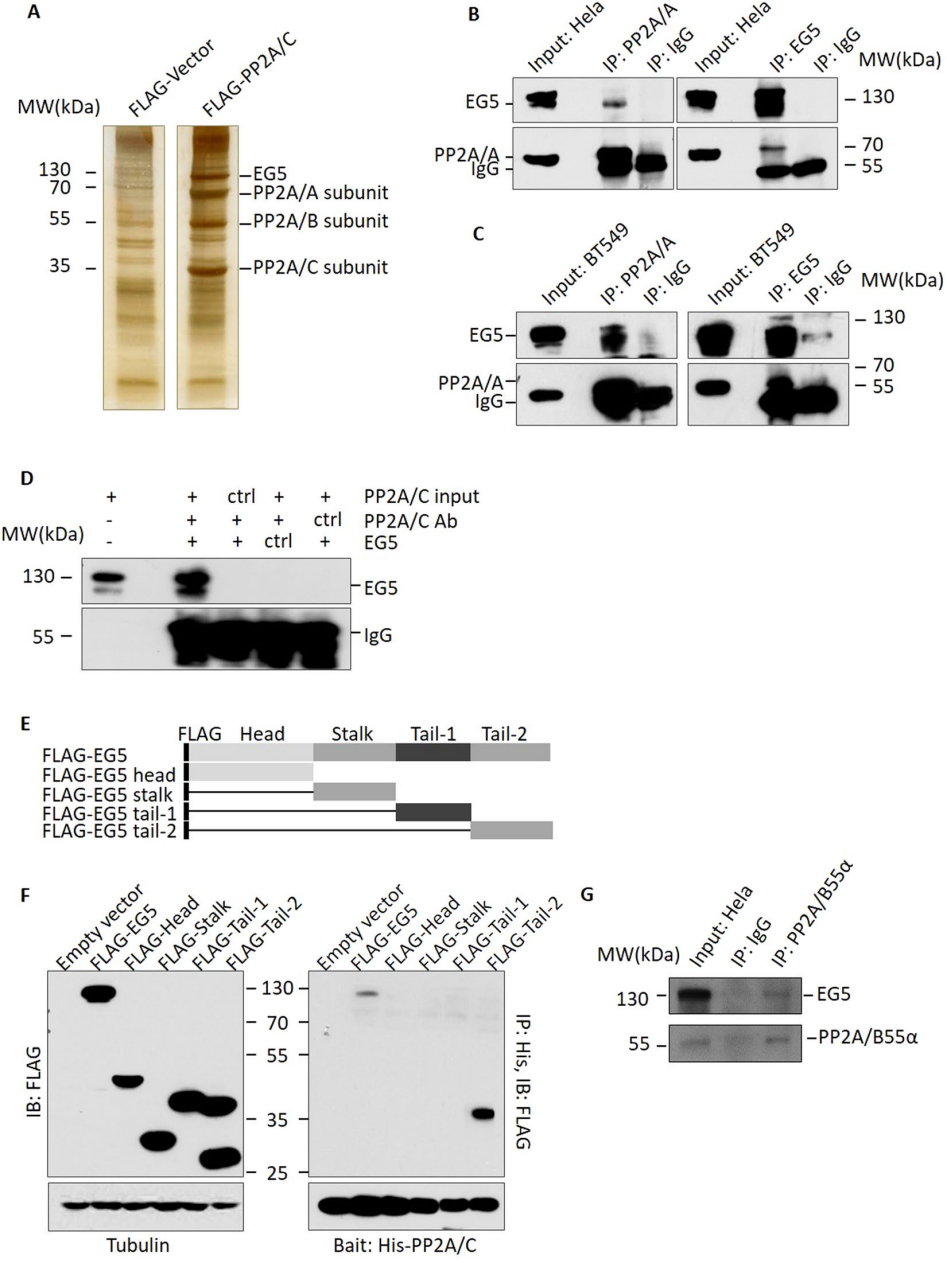
EG5 is a novel PP2A binding protein. (**A**) Silver staining of FLAG pull down assay. HeLa cells stably expressing FLAG-PP2A/Cα and control cells were lysed and incubated with FLAG antibody bound to beads, and FLAG peptide elutes were subjected to electrophoresis. (**B**,**C**) PP2A and EG5 showed association in HeLa and BT549 cells. Cell lysate was subjected to immunoprecipitation with anti-PP2A/A or anti-EG5 antibody, then evaluated with anti-EG5 or anti-PP2A/A antibody. (**D**) PP2A and EG5 interact *in vitro*. Purified His-PP2A/Cα was incubated with FLAG-EG5 from SF9 cells or with other control proteins, and the immunoprecipated complexes were identified with anti-FLAG antibodies. (**E**) Constructs of FLAG-tagged EG5 fragments (Head 1 ~ 350aa, Stalk 351 ~ 550aa, Tail-1551aa ~ 750aa, and Tail-2 751 ~ 1057aa). (**F**) The C terminal (Tail-2) of EG5 interacts with PP2A/C.EG5 and fragments expressed in HEK293T cells were incubated with His-PP2A/C, and precipitated with Ni-Beads. Association was evaluated with anti-FLAG antibody and Western blot. (**G**) EG5 associates with the PP2A/B55α complex. HeLa cell lysate was subjected to immunoprecipitation with anti-PP2A/B55α antibody, and evaluated with anti-EG5 and anti-PP2A/C antibodies. For the original uncropped western blots and gel data, see Supplementary information.

Slight decreases in the quantity of PP2A were observed to significantly delay metaphase-anaphase transition. In order to better characterize the role of PP2A in metaphase, we sought to determine which proteins associate with PP2A by blocking HeLa cells at metaphase with the microtubule inhibitor Nocodazole. FLAG-tagged PP2A/Cα was overexpressed in HeLa cells and then concentrated using beads conjugated to anti-FLAG antibody. In addition to the PP2A A, B, and C subunits, an unexpected band that was larger than any of the PP2A subtypes was observed, and was subjected to mass spectroscopy analysis (Fig. 2A). Use of the Mascot Server demonstrated 12.5% of the peptide sequences in this band were comprised of EG5 (peptides are listed in Supplementary Figure S1A). To confirm that PP2A interacts with EG5 *in vivo*, PP2A and EG5 were immunoprecipitated from a panel of human cell lines that were synchronized at metaphase. As expected, in both HeLa (Fig. 2B) and BT549 human breast carcinoma cells (Fig. 2C), endogenous EG5 effectively co-precipitated with PP2A, indicating these two proteins associate *in vivo*. An identical pattern was also observed in MB231 and MB435 human breast carcinoma cells (Supplementary Figure S1B). To confirm interaction of PP2A and EG5 *in vitro*, purified His-tagged PP2A/Cα and Flag-tagged EG5 were individually overexpressed in SF9 insect cells and incubated together, and EG5 and PP2A/Cα did in fact show *in vitro* association (Fig. 2D). Moreover, as EG5 increased, increases in PP2A/C were observed, implying that interaction of EG5 and PP2A/C is dose-dependent (Supplementary Figure S1C). We therefore concluded that PP2A/Cα interacts directly with EG5.

To determine the region of EG5 that interacts with PP2A, a series of FLAG-tagged EG5 fragments were constructed and expressed in HEK293T human embryonic kidney cells (Fig. 2E and F left panel). FLAG-tagged EG5 fragments were incubated with purified His-PP2A/Cα conjugated to Ni-NTA agarose. Immunoblot of the proteins precipitated with anti-FLAG antibody indicated that PP2A/Cα interacts only with full length EG5, or with the distal-most portion of the EG5 C-terminal tail (hereafter referred to as “Tail2”) (Fig. 2F, right panel). We therefore concluded that the site of interaction of PP2A/Cα and EG5 resides in Tail2 between residues 751 and 1057.

We next sought to determine which specific PP2A B-subunit(s) interacts with EG5 using a Flag pull-down assay with HEK293 cells expressing Flag-tagged Tail2. A band around 55 kD (Supplementary Figure S1D) was identified as the B55α subunit of PP2A by mass spectroscopy, and interaction of PP2A/B55α and EG5 was confirmed by IP Western blot in HeLa cells (Fig. 2G). B55α has been reported to be a key regulator of mitotic exit^30, 32^, which is consistent with our observations in shPP2A/C-2 Hela cells. Although B55α was the only B subunit identified in these results, the possibility that this may not be the only B subunit which interacts with EG5 cannot be excluded. Based on these results, we conclude that EG5 associates with PP2A by direct interaction with PP2A at the Tail 2 section of its C-terminal tail, and conclude B55α may also contribute to this interaction.

### PP2A dephosphorylates EG5 at Threonine 926

**Figure 3:**
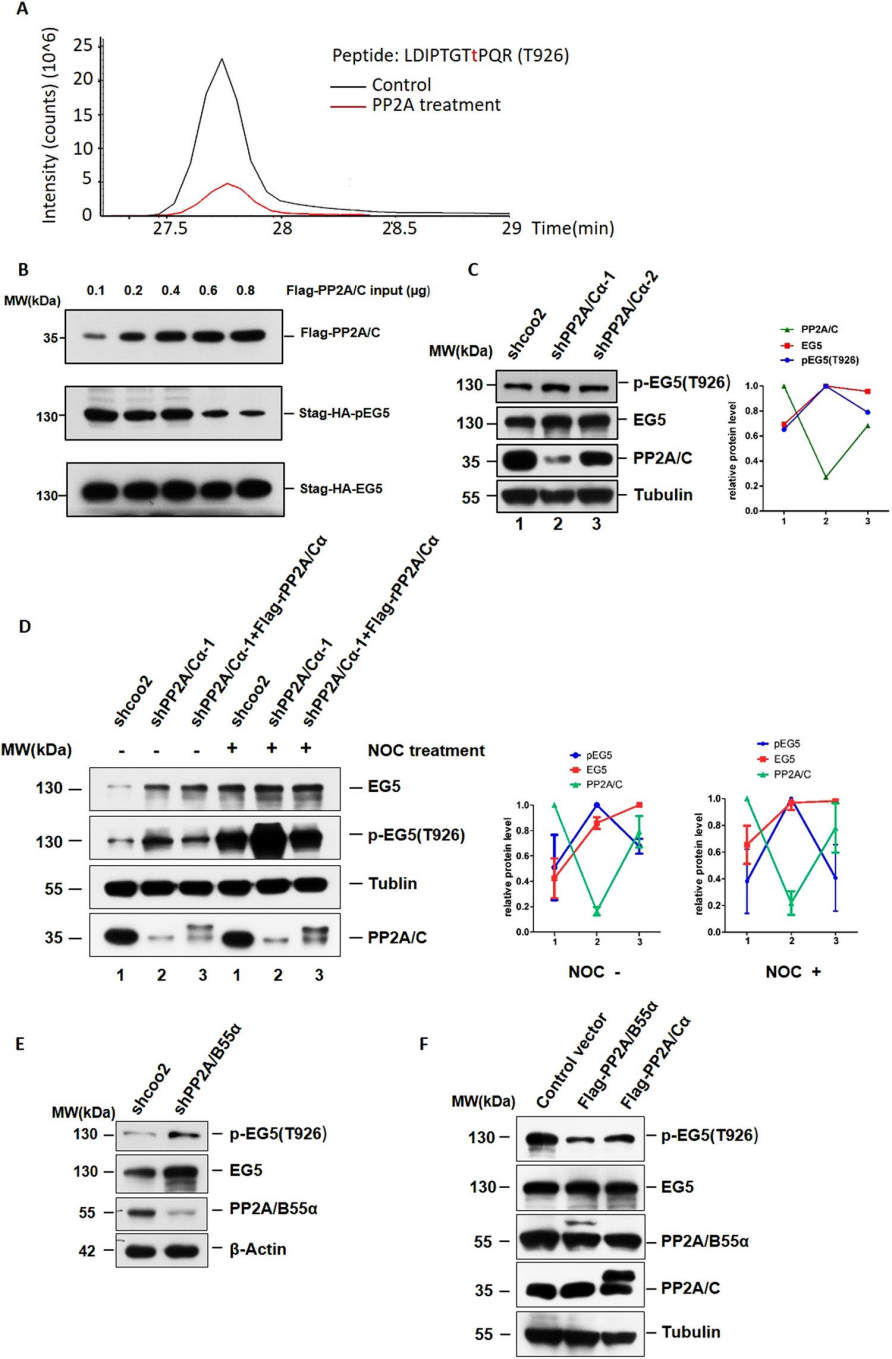
PP2A dephosphorylates EG5 at Threonine 926. (**A**) Intensity of peptide containing the phosphorylated Thr 926 site in PP2A/C or control protein treated EG5. (**B**) PP2A/C dephosphorylated EG5 in a concentration-dependent manner. Indicated amounts of His-PP2A/Cα were titrated into a constant amount of FLAG-EG5, reacted in phosphatase buffer at 30 °C for 30 minutes, and the mixture was precipitated using anti-FLAG antibody and subjected to Western blot. (**C**) Phosphorylation levels of EG5 (T926) in PP2A/Cα knockdown and control cells. (**D**) Protein levels of PP2A/C and phosphorylation level of EG5 (T926D) in HeLa cells with or without NOC treatment. rPP2A/Cα represents shPP2A/Cα resistant PP2A/Cα. (**E**) Phosphorylation levels of EG5 (T926) in PP2A/B55α knockdown and control cells. (**F**) Phosphorylation levels of EG5 (T926) and protein levels of PP2A/C or PP2A/B55α in HeLa cells overexpressing PP2AC/α or PP2A/B55α respectively. For the original uncropped western blot data, see Supplementary information.

As PP2A is an important phosphatase in eukaryotic cells, we posited the interaction of PP2A and EG5 may result in dephosphorylation of EG5. Purified EG5 was reacted with His-PP2A/Cα or control lysate in a phosphatase assay buffer for 30 minutes at 30 °C. EG5 was then segregated by SDS-PAGE electrophoresis, excised from the gel, and digested for identification of phosphorylation sites by mass spectrometry, and alterations in the EG5 phosphorylation level (Supplementary Figure S2). Among all EG5 phosphorylation sites identified, only threonine at 926 showed significant phosphorylation in the control group, which decreased dramatically after PP2A/Cα treatment (Supplementary Table S1). All changes identified in phosphorylation levels of phosphorylated EG5 sites can be found in Supplementary Table S1. The extent of phosphorylation of the modified peptide (LDIPTGTtPQR) containing phosphorylated Thr926 decreased markedly after PP2A treatment (Fig. 3A). To verify this site is a PP2A EG5 phosphorylation site, we performed dephosphorylation assays *in vitro* holding the level of EG5 constant and increasing PP2A/Cα, using specific anti-phospho-EG5 (T926) antibody. As the level of PP2A/Cα increased, the level of T926 phosphorylated EG5 (pEG5) decreased gradually, arguing PP2A dephosphorylates EG5 in a dose-dependent manner *in vitro* (Fig. 3B). Knock down of expression of the PP2A/Cα subunit in HeLa cells resulted in increases in the level of pEG5 compared with the level of pEG5 in scramble shRNA treated cells (Fig. 3C). Quantitative analysis showed that total EG5 was also negatively regulated by PP2A, although total EG5 was not regulated as stringently as pEG5 under changes in the amount of PP2A. These increases in pEG5 levels were reversed by introduction of Flag-shPP2A/Cα resistant PP2A/Cα (Flag-rPP2A/Cα) (Fig. 3D). This experiment was performed several times with both synchronized cells and cells grown asynchronously. It seemed that there were no significant differences in the reverse of pEG5 level caused by introduction of Flag-shPP2A/Cα resistant PP2A/Cα between the cells which were synchronized and the cells grown asynchronously. Over expression of PP2A/Cα in HeLa cells (Fig. 3F) consistently resulted in a decrease in pEG5. B55α (Fig. 2G) was identified as an EG5 associated PP2A subunit, and B55α protein levels affected EG5 phosphorylation status in the same manner as PP2A/Cα (Fig. 3E,F). We therefore concluded that both the PP2A/Cα and B55α subunits contribute to dephosphorylation of EG5, and the target site is Thr926.

The association of regulatory B subunits with the PP2A core enzyme achieves proper functioning and regulation of PP2A^33^. It has been reported that the assembly of the complex with the appropriate B-type subunit is the key to specificity and regulation of PP2A^34^. In order to further confirm the importance of B55α for the specificity of PP2A for EG5, we knocked down several B subunits (2R2B and 2R3A) in HeLa cells to compare the dephosphorylation of EG5 with these different B subunits. Knock down of 2R2B or 2R3A of PP2A did not result in increases in the pEG5 level compared with the level of pEG5 in scramble shRNA treated cells (Supplementary Figure S3).

### PP2A associates with EG5 localization during mitosis

**Figure 4:**
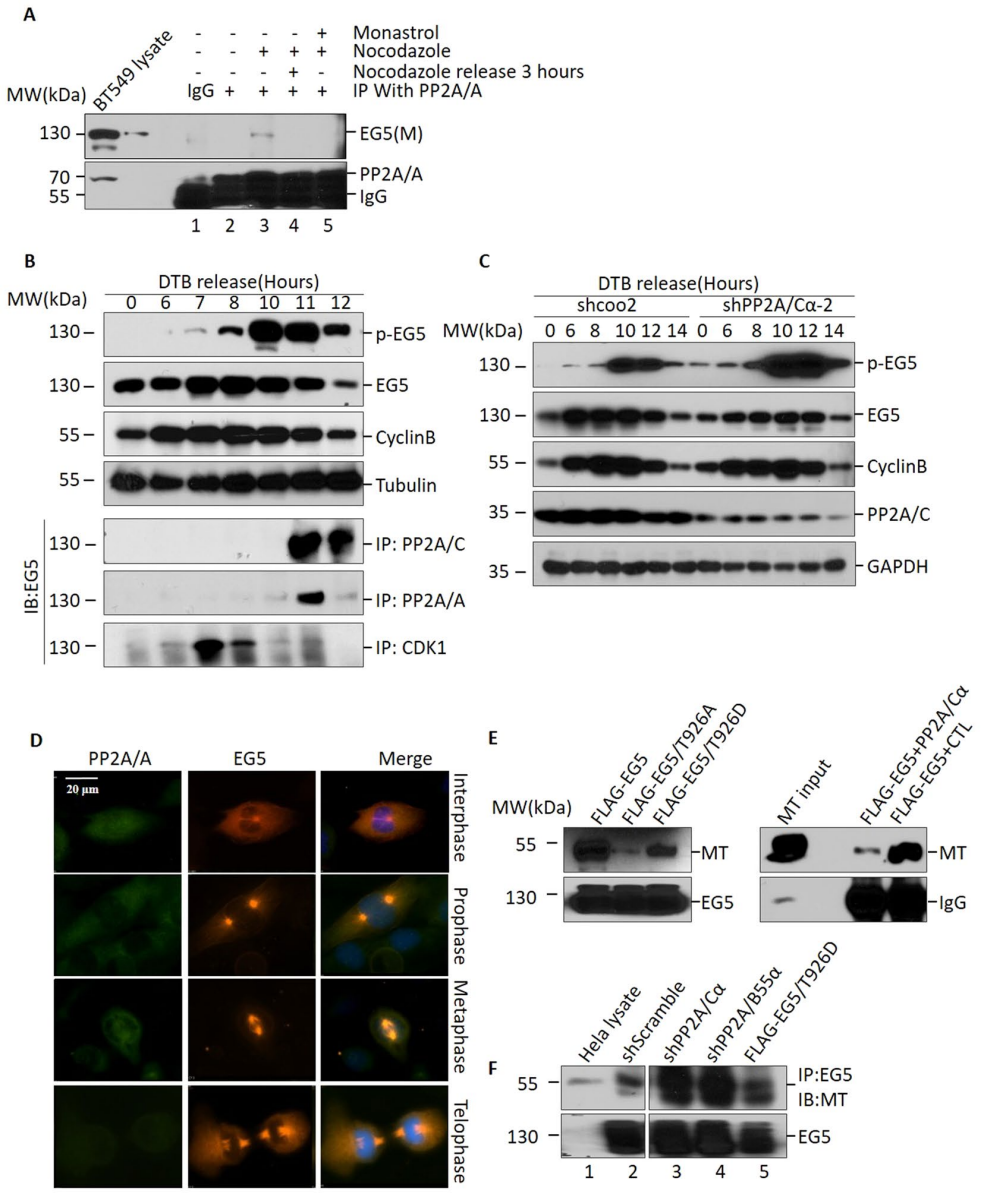
PP2A associates with EG5 localization during mitosis. (**A**) Co-immunoprecipitation of EG5 with PP2A/A after Nocodazole treatment. BT549 cells were treated with Nocodazole or Monastrol as indicated, then subjected to immunoprecipitation with anti-PP2A/A antibody followed by Western blot using anti-EG5 antibody. (**B**) HeLa cells were synchronized at G1/S with a double thymidine block (DTB), and were then released into fresh medium and harvested at indicated time points. Cell extracts were subjected to Western blot for evaluation of protein levels of phosphorylated EG5 (T926), total EG5 and cyclin B. Tubulin was used as loading control (upper panel). These cells were also subjected to co-immunoprecipitation with anti-PP2A/A, anti-PP2A/C and anti-CDK1 antibodies, and were then evaluated with anti-EG5 antibody. (**C**) HeLa cells infected with shPP2A/Cα-2 knockdown lenti-virus or control virus were synchronized with DTB and released into fresh medium. Whole cell lysate was harvested at indicated time points, then subjected to Western blot using anti-pEG5 (T926), anti-EG5, anti-cyclin B, anti-PP2A/C, or anti-tubulin antibodies. (**D**) Subcellular localization of PP2A/A and EG5 during mitosis in BT549 cells. Anti-EG5 and anti-PP2A/A antibodies were used to label endogenous EG5 and PP2A/A. DAPI was used to show chromatin status. Bar represents 10 μm. (**E**) Microtubule (MT) binding of EG5 and variants (left panel). MT purified from mouse brain was co-immunoprecipated with EG5 and its variants (T926A, T926D), then subjected to Western blot to evaluate the MT binding ability of EG5 and its variants. MT binding ability of EG5 decreased after PP2A/Cα treatment (right panel). (**F**) MT binding of endogenous EG5 in HeLa cells with decreased PP2A/Cα or PP2A/B55α, or overexpressed FLAG-EG5/T926D. For the original uncropped western blot data, see Supplementary information.

There is interaction of PP2A and EG5 in HeLa cells as shown in Fig. 2B. To further investigate the mechanism of EG5 dephosphorylation by PP2A, we evaluated interaction of PP2A and EG5 in HeLa cells with or without Nocodazole treatment, or with Nocodazole treatment followed by cell harvest 3 hours after Nocodazole release. Interaction between PP2A/A and EG5 was detected only in cells with Nocodazole treatment (Fig. 4A, lane 3). This result suggested that PP2A interacts with EG5 only during mitosis. In addition, this interaction is abolished by Monastrol (an EG5 inhibitor) even when cells are blocked at mitosis, which indicates EG5 activity is necessary for interaction of PP2A and EG5 (Fig. 4A, lane 5). Based on these results, we conclude PP2A interacts with EG5 during mitosis.

Mitosis is a complex phase of the cell cycle in which protein expression must be accurately coordinated for proper progression. To further characterize the interaction of PP2A and EG5 during mitosis, a double thymine block (DTB) was employed to further evaluate the mechanism of EG5 activity in the cell cycle. EG5 associated with PP2A 11 hours after DTB release coincident with the onset of cyclin B decrease, indicating mitosis was approaching an end (Fig. 4B). As a control, the EG5 kinase CDK1 showed interaction with EG5 6 to 8 hours after DTB release, resulting in phosphorylation of EG5 at Thr926 (Fig. 4B, bottom panel). Although the overall level of EG5 decreased over hours 11 and 12 after DTB release, the level of EG5 binding to PP2A/A and PP2A/C in late mitotic phase increased, indicating that PP2A interacts with EG5 as mitosis progresses, but does not depend on the level of EG5 or pEG5. It therefore appears that the *in vivo* and *in vitro* mechanisms of PP2A binding with EG5 are different. The interval over which EG5 dephosphorylation occurred coincided exactly with PP2A binding of EG5. It has been shown that CDK1 phosphorylates EG5 at Thr926, which promotes EG5 localization on the mitotic spindle and drives bipolar spindle formation via centrosome separation. To avoid the complication of CDK1 activity and timing of mitotic exit, we used RO-3306 to inhibit CDK1 activity after synchronizing control and PP2A/Cα depleted cells in mitosis, and then measure pEG5 levels. It showed that RO-3306 did not significantly influence the increase of pEG5 level caused by PP2A/Cα knockdown (Supplementary Figure S4). These results suggested that CDK1 did not play a key role in the elevation of p-EG5 level in late mitosis and raised the possibility that EG5 dephosphorylation in late mitosis is related to its interaction with PP2A.

As PP2A dephosphorylates EG5 11 hours after DTB release, we examined pEG5 levels after PP2A/Cα knockdown with shPP2A/Cα-2 at this specific time point. PP2A knockdown cells showed an extremely high level of pEG5 10 to 12 hours after DTB release as compared with scramble shRNA, while only limited increases in pEG5 were observed at other time points (upper panel of Fig. 4C). At the same time, cyclin B levels in the PP2A/Cα knockdown group and control group showed no differences during mitotic progression, suggesting that the increase in the EG5 phosphorylation level is not a result of increased mitotic duration or mitotic index, but is rather a result of a defect in dephosphorylation by PP2A.

To confirm that PP2A and EG5 co-localize in metaphase, we performed immunofluorescent analysis with BT549 cells during mitosis. PP2A localized mainly in the cytoplasm in interphase, prometaphase and anaphase, while in metaphase it showed obvious co-localization with EG5 at the spindle (Fig. 4D). Antibody control for Fig. 4D was also shown in supplementary information (Supplementary Figure S5). This observation is consistent with our finding that PP2A and EG5 associate mainly in late mitosis.

Microtubular localization and hydrolysis of ATP to obtain energy are two essential requirements for functional EG5 locomotion along microtubules. To determine how PP2A affects EG5 function, we investigated the effect of phosphorylation status on ATPase activity. T926D or T926A EG5 mutations were designed to mimic EG5 phosphorylation or dephosphorylation status respectively. Both of these EG5 mutations showed ATPase activity equal to wild type EG5 (Supplementary Figure S6A), which means alteration of phosphorylation status does not affect EG5 ATPase activity. An identical result was observed with PP2A/Cα treated EG5, which showed that ATPase activity was equal to that of control lysate treated EG5 (Supplementary Figure S6B). EG5 microtubule binding was evaluated with a microtubule pelleting assay as previously described^35^. Microtubules were isolated from mouse brain, and incubated with purified Flag-tagged EG5, EG5/T926A or EG5/T926D. EG5/T926A bound far fewer microtubules than EG5 alone or EG5/T926D (Fig. 4E, left panel). EG5 pre-incubated with PP2A bound fewer microtubules than EG5 pre-incubated with control protein (Fig. 4E, right panel). This result suggested that EG5 phosphorylation status is related to microtubule binding ability, and PP2A reduces EG5 microtubule binding. This assay was also performed using endogenous EG5. More microtubules bound to EG5 in both PP2A/Cα knockdown and PP2A/B55α knockdown HeLa cell lysate, as compared with the control cell lysate (Fig. 4F). We therefore conclude that the interaction with PP2A affects EG5 microtubule binding ability.

### Excessive phosphorylation of EG5 induced by PP2A deficiency causes metaphase delay and segregation error in anaphase

**Figure 5:**
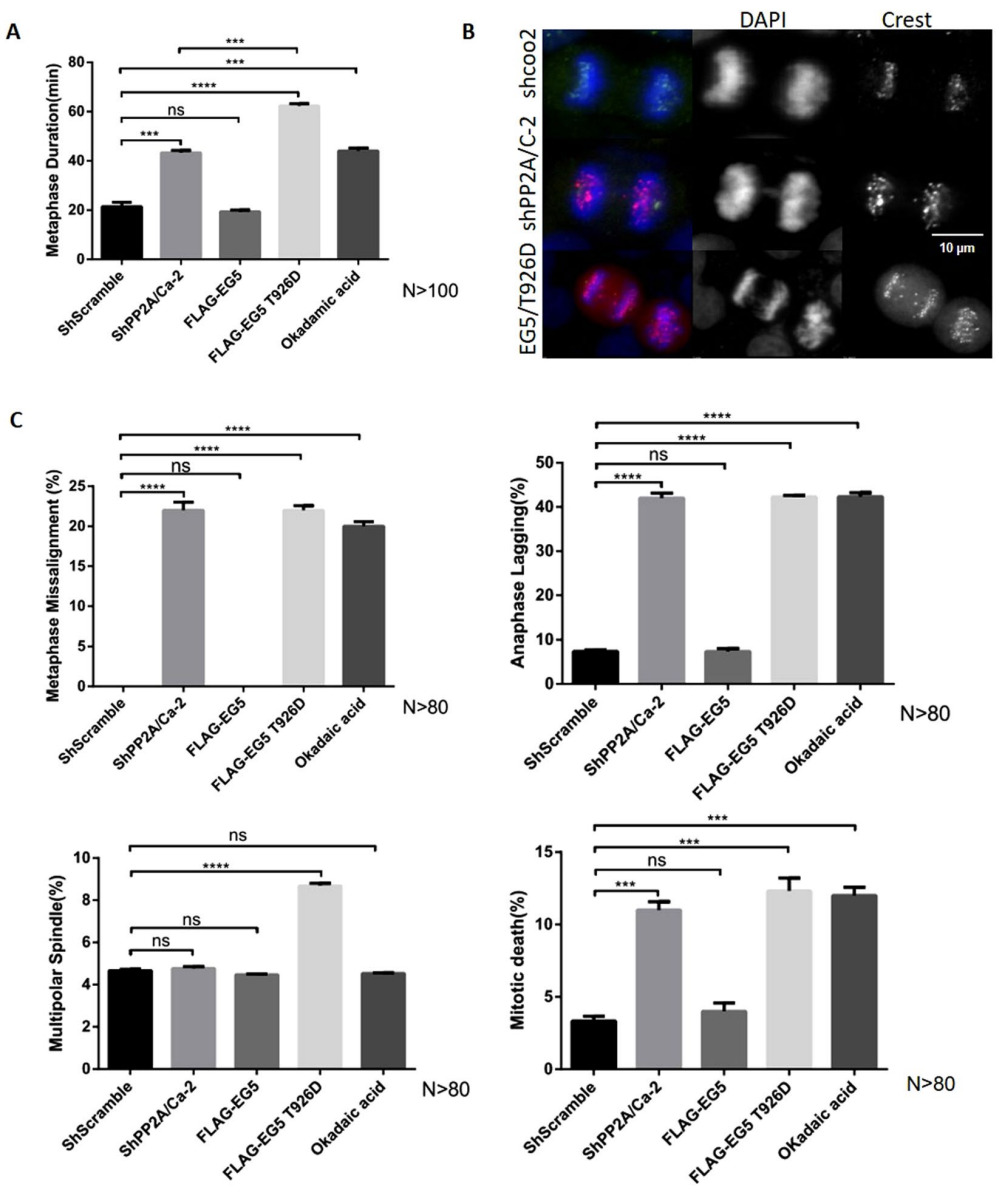
Excessive phosphorylation of EG5 induced by PP2A deficiency causes metaphase delay and segregation errors in anaphase. (**A**) Duration of metaphase in cells infected with lenti-virus (shcoo2, shPP2A/Cα-2), cells transfected with FLAG-tagged EG5, EG5 T926D and cells treated with Okadaic acid. (**B**) Duration of metaphase in HeLa cells overexpressing EG5T926D, or with PP2A/Cα knockdown, or with both over-expression of EG5 T926D and with PP2A/Cα knockdown. (**C**) A variety of mitotic errors occurred in HeLa cells (shcoo2, shPP2A/C-2, EG5, EG5/T926D, Okadaic acid) (N > 80).

PP2A deficiency results in EG5 over-phosphorylation in HeLa cells as demonstrated. In order to determine whether PP2A regulates metaphase progression through EG5, we first measured alteration of metaphase duration after PP2A knock down or with EG5/T926D overexpression. Overexpression of wild type EG5 (FLAG-tagged EG5) was also included as a control and showed no significant difference in shScramble cells. PP2A knock down increased metaphase duration from 22 to 50 minutes as previously observed, and EG5/T926D overexpression showed results similar to PP2A knockdown (Figs 1 D and 5A). In addition, we also used a low doses of okadaic acid to partially inhibit PP2A, and demonstrated these experimental results are reproducible (Fig. 5A). These results confirmed our hypothesis that PP2A regulates metaphase progression through EG5. In addition, the increase in mitotic index induced by PP2A knockdown was partially reversed by EG5/T926A overexpression (Supplementary Figure S7). However, although EG5/T926A reversed the effect of PP2A knockdown to some extent, metaphase duration was not restored to its original shorter length. This result suggested there are other factors in addition to EG5 through which PP2A regulates metaphase progression. Segregation errors in metaphase and anaphase were also obvious in both PP2A knockdown HeLa cells, and in cells overexpressing EG5/T926D. About 30% of cells showed defects of chromosomal alignment in metaphase, or chromosomal lagging at anaphase segregation (Fig. 5B,C). These errors affect chromosome integrity and may be transmitted to daughter cells, thus perpetuating errors in the next cycle of mitosis.

## Discussion

In this study, we identify EG5 as a novel substrate of protein phosphatase PP2A and show T926 is its major site of phosphorylation. A finding of particular significance is that PP2A dephosphorylation of EG5 occurs mainly in late metaphase and plays a role in mitotic exit. PP2A deficient cells show over-phosphorylation of EG5 (T926) especially at metaphase, while EG5 phosphorylation levels in control cells begin to decrease. As a consequence, delay in mitotic exit is observed in PP2A deficient cells. These results represent a novel mechanism by which PP2A regulates the location and function of the kinesin protein EG5, and provide evidence of the important role played by PP2A in the regulation of mitotic exit.

PP2A is one of the most important protein phosphatases in mammalian cells, and it takes part in almost all phases of mitosis. Efficient PP2A knockdown causes extensive cell death in HeLa cells. We therefore employed less vigorous knockdown in order to identify the phenotypes obscured by the extensive cell death brought about by efficient knockdown. This milder knockdown of PP2A caused delay of metaphase-anaphase transition and obvious delay of mitotic exit, arguing that in addition to the requirement for PP2A to simply be present, a specific quantity of PP2A is also critical for mitotic exit.

Typically the PP2A core enzyme exists as a dimer consisting of a catalytic subunit (C subunit/PP2AC) and a scaffolding A subunit (PR65/A subunit). It has been reported that the B-type subunit is the key to the specificity and regulation of PP2A^34^. Our data suggested that the PP2A B55α subunit contributes to the interaction of EG5 and PP2A *in vivo*. At the same time, we demonstrated that the PP2A/C subunit has the capability to bind with EG5 *in vitro* without the interaction of any other molecules (Fig. 2D). Therefore, we consider B55α as a facilitator of EG5 dephosphorylation. It has been reported that although a complex of PP2A/A and C subunits has full phosphatase activity *in vitro*, a holoenzyme consisting of all A, B, C subunits is the most common form of active PP2A phosphatase *in vivo*. The *in vivo* efficiency of a PP2A phosphatase without a B subunit does not compare with a phosphatase which has all three subunits^34, 36^. The B subunit plays an important role in the regulation of PP2A phosphatase activity with its substrate, and in addition, the correct specific type of B subunit is required for this regulation. Based on our observations, we propose that: a) the binding and dephosphorylation of EG5 occurs in all phases of the cell cycle, but the binding capability and efficiency of dephosphorylation likely vary from phase to phase; and b) Both binding and dephosphorylation are regulated not only by the phosphorylation status of EG5 and co-localization of PP2A and EG5, but also by the particular B subunit which participates in the formation of the PP2A holoenzyme in the reaction site. In this study, we have shown the B55α (but not 2R2B and 2R3A) subunit binds and facilitates the dephosphorylation of EG5. However, the possibilty that there are some other B subunits which also participate in the dephosphorylation of EG5 cannot be excluded. At this time, we have little specific information about when and how B subunits switch from one complex to another.

T926 and S1033 are two reported EG5 phosphorylation sites^27, 37^. Phosphorylation of T926 is linked to EG5 localization on the spindle, while S1033 is related to EG5 localization at the spindle pole^37^. Mass spectrum analysis was employed to assess phosphorylation level changes in EG5 phosphorylation sites after reaction with PP2A, and a significant reduction in T926 phosphorylation was found. However no phosphorylation at S1033 was detected with mass spectroscopy analysis. These results confirmed T926 is strongly phosphorylated compared to other EG5 phosphorylation sites, and suggests PP2A selectively regulates EG5 phosphorylation status during mitosis.

We observed PP2A associates with EG5 after treatment, when cells are synchronized at mitosis. However, this association is eliminated if EG5 function is abolished by Monastrol, which inhibits EG5 ATPase activity and prevents EG5 binding to microtubules^38^. These results indicate that functional EG5 is necessary for PP2A-EG5 interaction *in vitro*. Nonetheless, Monastrol inhibits EG5 by binding to its motor domain^39^, which resides well away from the Thr926 phosphorylation site with which PP2A interacts. Monastrol thus obstructs interaction between EG5 and PP2A through a mechanism other than competitive binding. It appears PP2A preferentially interacts with EG5 localized on the microtubule. At the same time, we observed that the amount of EG5 with which PP2A interacts is greater at 11 hours after DTB release than 10 hours after release, while pEG5 levels are nearly identical at these two time points. This means the association of EG5 and PP2A *in vivo* does not depend on the phosphorylation status of EG5. EG5 activation is thus required, but is not in itself sufficient for PP2A binding to EG5. There are likely other mechanisms which regulate PP2A and EG5 interaction at specific time points.

EG5 inhibition has been suggested as a potential cancer therapy, and several EG5 inhibitors have entered clinical trials (http://clinicaltrials.gov). STLC (S-Trityl-L-cysteine) is a specific inhibitor of EG5 that blocks EG5 ATPase activity and abolishes the ability of EG5 to bind microtubules. STLC is considered as a potential anti-mitotic chemotherapeutic agent^40^. We treated cells expressing EG5/T926D or cells defective in PP2A/B55α with STLC. Both groups of cells with excess phosphorylated EG5 were more resistant to EG5 inhibitor than control cells (Supplementary Figure S8), suggesting that EG5 inhibitors would be less effective against tumors where PP2A/C has been compromised. This study enlarges upon the scope of PP2A tumor suppressor function, and calls attention to the anti-neoplastic tolerance in PP2A defective patients. Even a small reduction in PP2A increases tolerance to EG5 inhibitors.

In this study EG5 is identified as a novel substrate of PP2A phosphatase, and we found that the PP2A/B55 complex plays an important role in mitotic exit by a mechanism involving EG5. The PP2A/B55 complex physically associates with the C-terminal tail domain of EG5, and dephosphorylates EG5 at Thr926 which enables mitotic exit. Moreover, PP2A knockdown cells show a high level of phospho-EG5 in late metaphase, associated with delay in mitotic exit. These phenotypic features are similar to those induced by EG5/T926D transfection, which mimics phosphorylated EG5 status. Our results argue PP2A controls mitotic exit through EG5 dephosphorylation. Lack of PP2A leads to the abnormal activation of EG5, resulting in delay of mitotic exit. EG5 is a cancer therapeutic target, and this study also calls attention to the anti-neoplastic tolerance in PP2A defective patients.

## Methods

### Flag-pull down and mass spectrum analysis

Lysates from HeLa cells over-expressing Flag-tagged PP2A/Cα were used to perform Flag-pulldown experiments, according to the instructions from the Sigma FlagHA-Pulldown kit. Eluted proteins were loaded onto SDS-page gels, silver stained, digested with trypsin, and subjected to mass spectrum sequencing (using an ESI-QUAD-TOF instrument). Results were analyzed using the Mascot Server (Matrix Science).

### Cell Culture and Constructs

All cancer cells were obtained from the American Type Culture Collection (ATCC), and cultured in MEM medium supplemented with 10% FBS (Atlanta Biologicals). SF9 insect cells were obtained from Invitrogen, and were cultured in Grace’s insect medium (Gibco) supplemented with 10% FBS. Double thymidine block was performed by treating HeLa cell with 2 mM Thymidine (Sigma) for 18 hours, followed by release for 8 hours, and reblocking with 2 mM thymidine for another 18 hours. Cells were then synchronized at G1/S phase. DNA sequences of PP2A subunits and EG5 used for plasmid construction were obtained by reverse transcription from HeLa cell mRNA.

### Protein purification

Proteins were expressed with the pFastBac1 eukaryotic expression system (Invitrogen) and the pET prokaryotic expression system (Novagen). Flag-tagged Protein was purified with ANTI-FLAG M2 Affinity Gel (Sigma), and His-tagged protein was purified with Ni-NTA Superflow beads (Qiagen).

### shRNA

The PLKO.1 system was used to knock down target genes. The plasmids PLKO.1 (Addgene plasmid 8453)^41^, pMD2.G(Addgene plasmid 12259)(Didier Trono Lab), and psPAX2 (Addgene plasmid 12260)(Didier Trono Lab) were obtained from Addgene. shRNA targeting sequences were as follows: PP2A/Cα shRNA1, AAGAGGTTCGATGTCCAGTTA^30^; pp2ac-alpha shRNA2, AATGGAACTTGACGATACTCTA; PP2A/B55αshRNA, CTGCAGATGATTTGCGGATTA.

### Phosphatase Assay

Flag-EG5 and His-PP2A protein were purified from SF9 insect cells and incubated together for 30 mins at 30 °C in Phosphatase Aaady Buffer (20 mM Hepes pH 7.2, 1 mM DTT, 1 mM MgCl_2_, 0.1 mg/ml BSA, 1 mM EDTA), and were then used for mass spectrometric analysis or Western Blot.

### Sample Preparation and MS Analysis

Following electrophoresis, bands were excised, and gel digestion was performed according to standard procedures^42^. Phosphopeptides were enriched using chitosan-GMA-IDA-Fe(III) nanospheres as previously described^43^. Digests were redissolved and injected into an EASY-nLC 1000 instrument, and analyzed using a 60 min elution program. Spectra were acquired with an LTQ OrbitrapVelos pro mass spectrometer (Thermo Fisher Scientific, Bremen, Germany). Proteome Discoverer 1.3 software was used for data analysis in the IPI human v3.83 database. Label-Free Phosphopeptide quantification was carried out as previously described^44^ with minor changes. All phosphopeptides containing the phosphorylated Thr926 site were summed to determine the total level of phosphorylation.

### Immunofluorescence

BT549 cells were grown on cover slides. Cells were rinsed with PBS, fixed with 4% formaldehyde, and permeabilized with 1% Triton X-100. Cells were then blocked with 1% BSA, and incubated with anti-PP2A/A antibody which reacts with both alpha and beta subunits (SantaCruz)(1:200) and anti-EG5 antibody (Biolegend)(1:200) overnight at 4 °C in a humidified chamber. After washing with PBS, cells were incubated for 1 h at room temperature with appropriate secondary antibody. Cells were washed with PBS before DAPI staining.

### Live cell imaging

HeLa cells expressing GFP-H2B and mcherry-α-tubulin were infected with scrambled shRNAlenti-virus or shPP2A shRNA. 48 hours after infection, cells were placed in an 8-well chamber with a glass bottom (BD). The medium was changed to phenol red free medium 4 hours prior to microscopic evaluation (Nikon DS-U3). The environmental chamber was kept humid with a 5% CO_2_ atmosphere. For long-term imaging, pictures were taken every 3 minutes for 72 hours.

### Immunoprecipitation

For analysis of PP2A and EG5 interaction, HeLa cells or BT549 cells were treated with 100 nM Nocodazole for 5 hours and lysed in NP-40 lysis buffer. Antibodies to PP2A/C subunits (Cell signaling), PP2A/A subunits (Santa Cruz), EG5 (Biolegend), or CDK1 (Santa Cruz), were incubated with cell extracts at 4 °C for 16 hours. Antibody-protein complex was bonded to Protein A/G beads and then subjected to Western Blot after washing 3 times with NP40 lysis buffer.

### ATPase assay

Purified EG5 or EG5 mutations treated with PP2A phosphatase or control proteins were incubated with ATP substrate at 25 °C for 30 minutes. PiColorLockTM Gold reagent (Novus) was then added into the reaction mixture to evaluate free Pi released from EG5 using a spectrophotometer.

### Microtubule pelleting assay

A pelleting assay was performed in buffer (20 mM PIPES, pH 6.9, 1 mM DTT, and 5 mM MgCl_2_) supplemented with taxol (10 uM). Flag-beads bound to EG5 derived from various cell clones or treatments were added to Taxol stabilized MTs in a total volume of 200 ul. Samples were gently mixed and incubated for 15 min at 25 °C. Flag beads were then spun down and subjected to Western Blot. Anti-α-tubulin antibody (Abcam) was used to evaluate the capacity of EG5 for binding to MTs.

### EG5 phosphorylation site sequencing

Flag-EG5 purified from SF9 was incubated with SF9 insect cell lysate containing overexpressed His-PP2A protein or control for 30 mins at 30 °C in phosphatase assay buffer (20 mM Hepes pH 7.2, 1 mM DTT, 1 mM MgCl_2_, 0.1 mg/ml BSA, 1 mM EDTA). EG5 was then concentrated on Flag-beads and subjected to SDS-PAGE gel electrophoresis. Trypsin digestion was performed at 37 °C overnight. Phosphopeptides were enriched with Ti4+-IMAC microspheres. Mass spectrometric analysis was carried out following a previously described protocol^45^.

### MTT assay

Cells infected with various shRNAs were plated in 96-well plates for assay with 3-(4,5-dimethyl-2-thiazolyl)-2,5-diphenyl 2H-tetrazolium bromide (MTT, Sigma). STLC (2 µM) was added into wells after 12 hours. 20 μL of 5 mg/mL MTT was added to each well after 24, 48, 72 and 96 hours. Cells were incubated with MTT at 37 °C for 4 hours, after which the supernatant was removed carefully. 150 μL of dimethyl sulfoxide (DMSO) was added to each well. The OD of each well was measured at 490 nm after 10 minutes. All experiments were performed in triplicate.

## Electronic supplementary material

SREP-15-29701B-Supplementary Information
