# Supplementary material for: Protein Phosphatase 2A (PP2A) Regulates EG5 to Control Mitotic Progression
Source: Sci Rep. 2017 May 9;7:1630. doi: 10.1038/s41598-017-01915-w (PMC5431654; doi:10.1038/s41598-017-01915-w)
Supplement: Supplementary file 1 — SREP-15-29701B-Supplementary Information [file 41598_2017_1915_MOESM1_ESM.doc]

**Protein Phosphatase 2A (PP2A) Regulates EG5 to Control Mitotic Progression**

Yang Liu^1, 3^, Zhong Zhang^1^, Hui Liang^1^, Xuyang Zhao^1^, Ling Liang^1^, Guangxi Wang^1^, Jingyi Yang^1^, Yan Jin^1^, Michael A. McNutt^1^, &Yuxin Yin^1, 2, 3*^

**Supplementary**

**Figure S1**

1. Sequence coverage of EG5: 12%

MASQPNSSAK KKEEKGKNIQ VVVRCRPFNL AERKASAHSI VECDPVRKEV

51 SVRTGGLADK SSRKTYTFDM VFGASTKQID VYRSVVCPIL DEVIMGYNCT

101 IFAYGQTGTG KTFTMEGERS PNEEYTWEED PLAGIIPRTL HQIFEKLTDN

151 GTEFSVKVSL LEIYNEELFD LLNPSSDVSE RLQMFDDPRN KRGVIIKGLE

201 EITVHNKDEV YQILEKGAAK RTTAATLMNA YSSRSHSVFS VTIHMKETTI

251 DGEELVKIGK LNLVDLAGSE NIGRSGAVDK RAREAGNINQ SLLTLGRVIT

301 ALVERTPHVP YRESKLTRIL QDSLGGRTRT SIIATISPAS LNLEETLSTL

351 EYAHRAKNIL NKPEVNQKLT KKALIKEYTE EIERLKRDLA AAREKNGVYI

401 SEENFRVMSG KLTVQEEQIV ELIEKIGAVE EELNRVTELF MDNKNELDQC

451 KSDLQNKTQE LETTQKHLQE TKLQLVKEEY ITSALESTEE KLHDAASKLL

501 NTVEETTKDV SGLHSKLDRK KAVDQHNAEA QDIFGKNLNS LFNNMEELIK

551 DGSSKQKAML EVHKTLFGNL LSSSVSALDT ITTVALGSLT SIPENVSTHV

601 SQIFNMILKE QSLAAESKTV LQELINVLKT DLLSSLEMIL SPTVVSILKI

651 NSQLKHIFKT SLTVADKIED QKKELDGFLS ILCNNLHELQ ENTICSLVES

701 QKQCGNLTED LKTIKQTHSQ ELCKLMNLWT ERFCALEEKC ENIQKPLSSV

751 QENIQQKSKD IVNKMTFHSQ KFCADSDGFS QELRNFNQEG TKLVEESVKH

801 SDKLNGNLEK ISQETEQRCE SLNTRTVYFS EQWVSSLNER EQELHNLLEV

851 VSQCCEASSS DITEKSDGRK AAHEKQHNIF LDQMTIDEDK LIAQNLELNE

901 TIKIGLTKLN CFLEQDLKLD IPTGTTPQRK SYLYPSTLVR TEPREHLLDQ

951 LKRKQPELLM MLNCSENNKE ETIPDVDVEE AVLGQYTEEP LSQEPSVDAG

1001 VDCSSIGGVP FFQHKKSHGK DKENRGINTL ERSKVEETTE HLVTKSRLPL

1. RAQINL

1. **
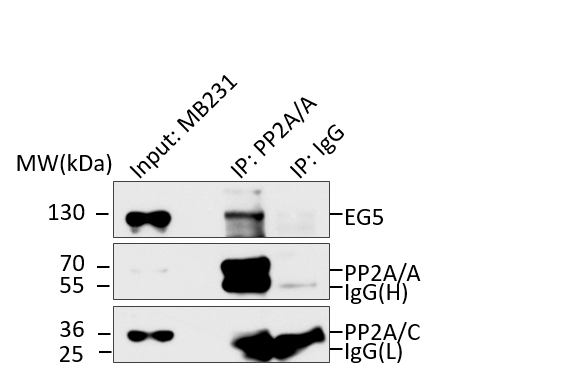

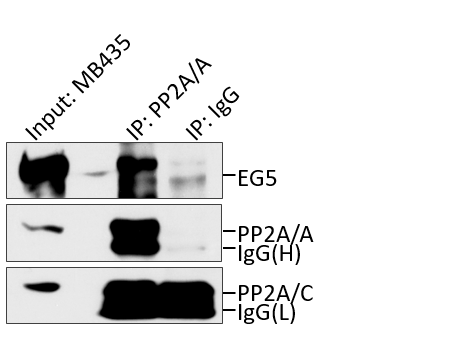
**

**
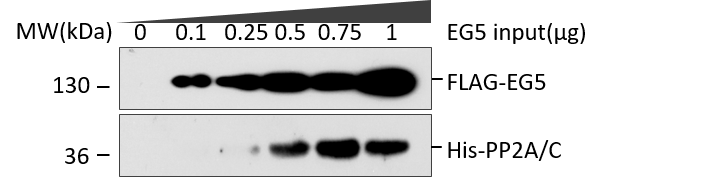
**

**
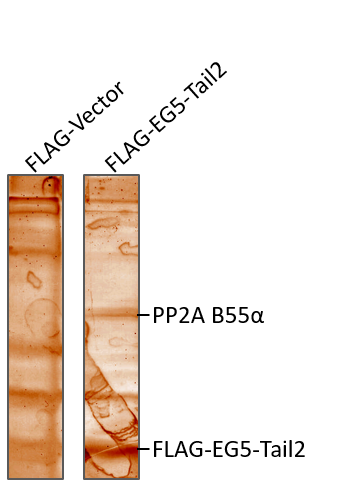
**

(A) Mass spectrometric sequence coverage of EG5 protein. (B) IP WB with anti-PP2A/A subunit antibody in MB231 or MB435 cell lysate. (C) EG5 associates with PP2A/C in a dose-dependent manner. Indicated amounts of FLAG-EG5 were incubated with a constant amount of His-PP2A/C, and then precipitated using anti-FLAG antibody. (D) Pull-down experiments were performed with anti-FLAG resins in HEK293T cell lysate which contained overexpressed FLAG-tagged EG5-Tail2. Silver staining showed protein bands that interact with the EG5-Tail2 fragment.

**Figure S2. Mass spectrometric signal identifying the phosphorylated site of EG5 (Thr926).**


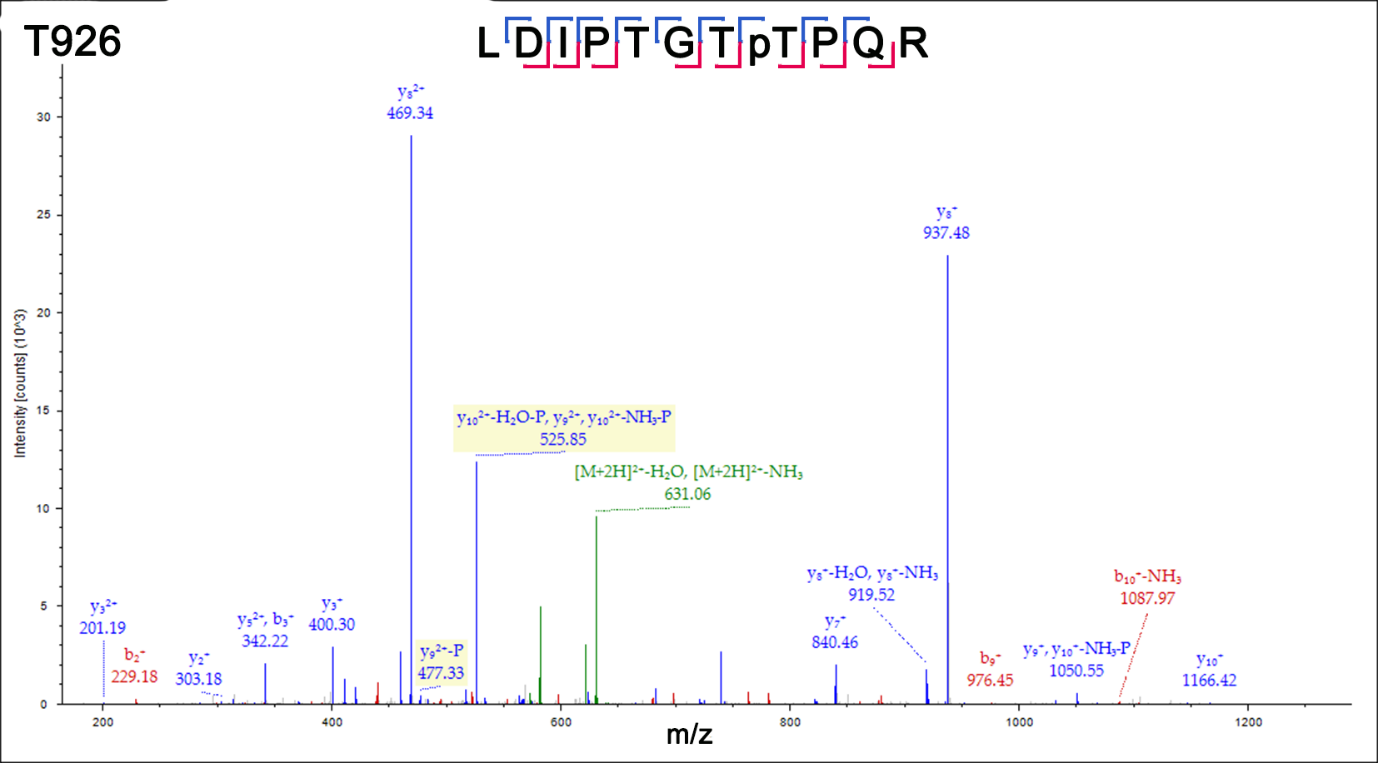


**
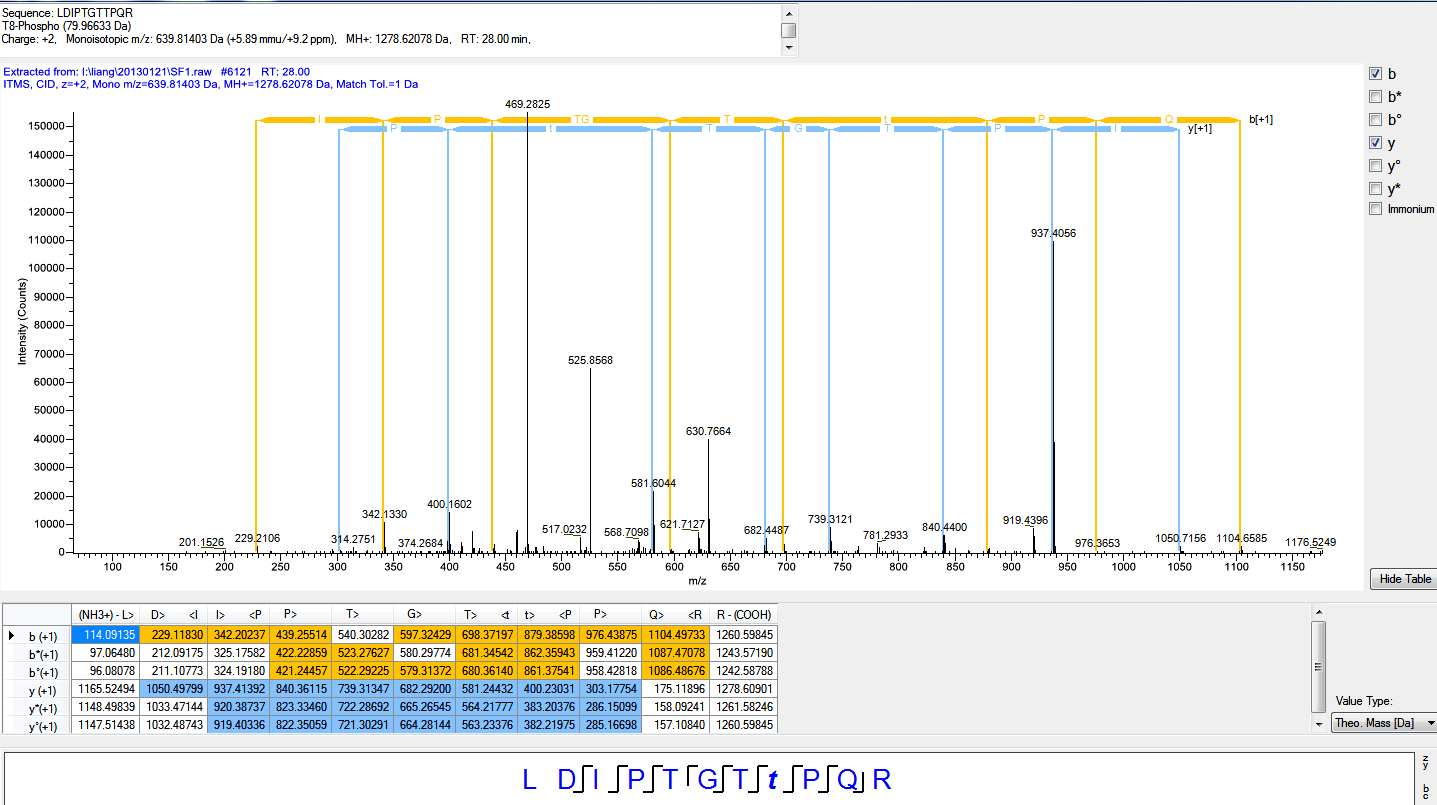
**

Mass spectrometric analysis identified the band as the phosphorylated site of EG5 (Thr926)

**Figure S3.**

**
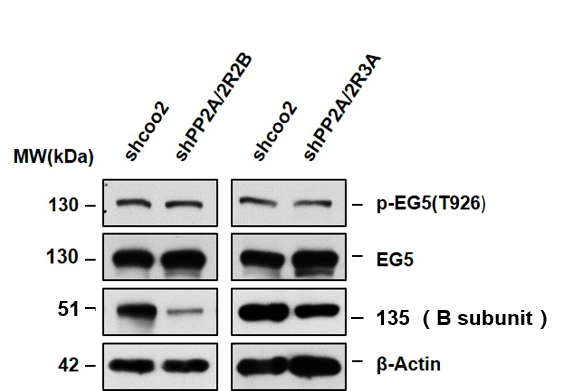
**

Phosphorylation levels of EG5 (T926) in PP2A/B subunits (2R2B and 2R3A) knockdown and control cells

**Figure S4.**

**
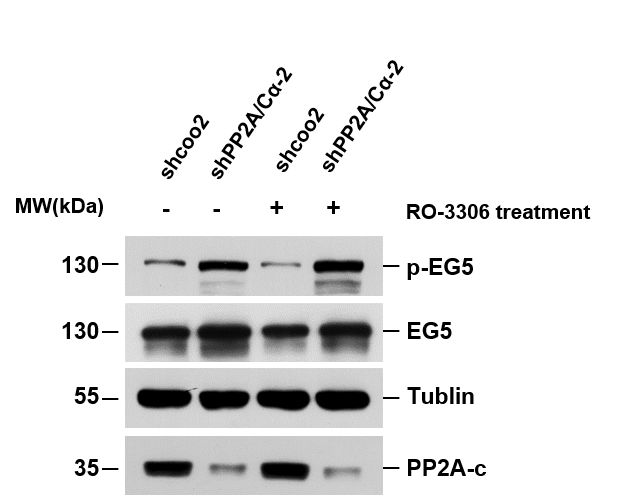
**

RO-3306 was used to inhibit CDK1 activity. Both PP2A/Cα knock down cells and control cells were synchronized to M phase with 16 hours of Nocodazole treatment, and then these cells were treated with or without RO-3306 for 2 hours followed by harvest and measurement of pEG5 levels.

**Figure S5. Antibody control for Figure4D**


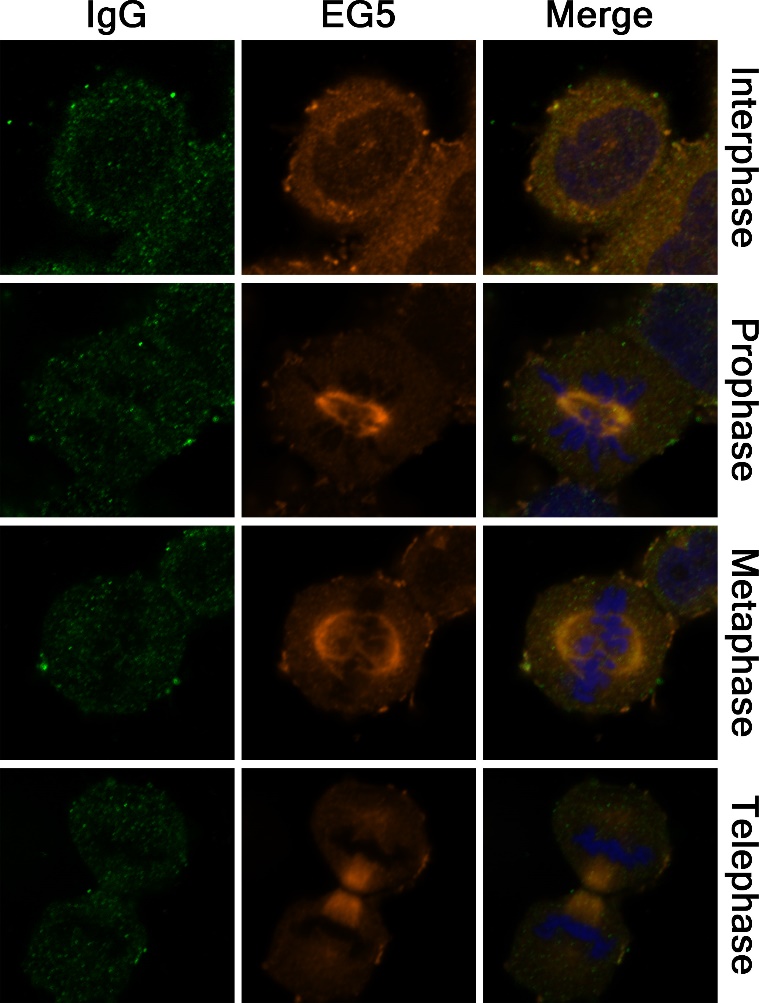


**10 μm**

Subcellular localization of EG5 (IgG) during mitosis in HeLa cells. Anti-EG5 antibodies was used to label endogenous EG5. DAPI was used to show chromatin status. Scale bar represents 10 μm.


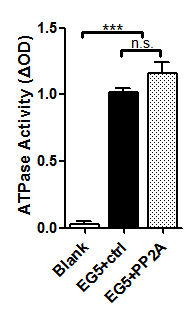
**Figure S6**


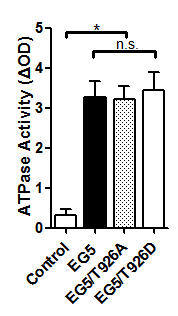
**A B**

(A) ATPase Activity of EG5 and EG5 mutations. EG5, EG5/T926A, EG5/T926D proteins were purified from the SF9 expression system, and measured with an ATPase assay. (B) ATPase Activity of EG5 with or without PP2A treatment. Purified EG5 proteins were incubated with PP2A or control protein in dephosphorylation buffer, and then applied to the ATPase assay.

**Figure S7. Duration of metaphase**

**
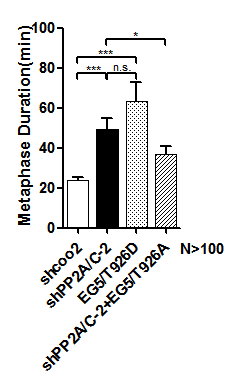
**

Duration of metaphase in cells infected with lenti-virus (shcoo2, shPP2A/Cα-2) and/or transfected with FLAG-tagged EG5 T926A/D.

**Figure S8. STLC inhibition curve according to MTT assay.**

**
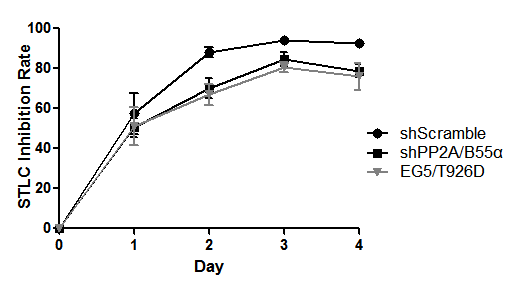
**

STLC (EG5 inhibitor) inhibition curve according to the MTT assay (Bars represent ± SEM).

**Table S1. All changes identified in phosphorylation levels of phosphorylated sites of EG5.**

**Intensity of p-peptide of EG5 (10^5^ Counts)**

| Phospho-site | shPP2A/Cα-2 | shScramble |
| --- | --- | --- |
| S39 | 0.00 | 12.02 |
| S233 | 0.00 | 16.36 |
| S401 | 2.44 | 5.48 |
| T458 | 0.00 | 1.74 |
| S487 | 3.85 | 11.67 |
| S748 | 0.00 | 5.56 |
| S852 | 47.77 | 65.32 |
| T926 | 5911.17 | 14674.92 |
| S1033 | 0.00 | 0.00 |

**Table S2.A list of matched peptides from MS analysis**

| **Accession** | **Coverage** | **# PSMs** | **# Peptides** | **# AAs** | **MW [kDa]** | **calc. pI** | **Score** | **Description** |
| --- | --- | --- | --- | --- | --- | --- | --- | --- |
| **IPI:IPI00304925.5** | 40.72 | 33 | 21 | 641 | 70.0 | 5.66 | 1939.48 | Heat shock 70 kDa protein 1A/1B |
| **IPI:IPI00003865.1** | 38.54 | 32 | 20 | 646 | 70.9 | 5.52 | 1803.46 | Isoform 1 of Heat shock cognate 71 kDa protein |
| **IPI:IPI00911039.1** | 42.83 | 31 | 21 | 586 | 63.9 | 5.55 | 1746.27 | cDNA FLJ54408, highly similar to Heat shock 70 kDa protein 1 |
| **IPI:IPI00645452.1** | 44.60 | 19 | 12 | 426 | 47.7 | 4.81 | 915.33 | Uncharacterized protein |
| **IPI:IPI00966238.2** | 26.02 | 16 | 13 | 665 | 72.4 | 5.94 | 864.47 | cDNA FLJ51907, highly similar to Stress-70 protein, mitochondrial |
| **IPI:IPI00007752.1** | 39.33 | 17 | 11 | 445 | 49.8 | 4.89 | 858.11 | Tubulin beta-2C chain |
| **IPI:IPI00013475.1** | 32.81 | 16 | 10 | 445 | 49.9 | 4.89 | 799.79 | Tubulin beta-2A chain |
| **IPI:IPI00893099.1** | 22.78 | 15 | 10 | 641 | 70.4 | 6.29 | 749.82 | Uncharacterized protein |
| **IPI:IPI00023598.2** | 31.98 | 13 | 8 | 444 | 49.6 | 4.88 | 647.55 | Tubulin beta-4 chain |
| **IPI:IPI00975690.1** | 22.04 | 9 | 8 | 431 | 49.6 | 5.25 | 532.61 | Vimentin variant 3 |
| **IPI:IPI00009865.4** | 26.71 | 11 | 11 | 584 | 58.8 | 5.21 | 507.22 | Keratin, type I cytoskeletal 10 |
| **IPI:IPI00007702.1** | 8.29 | 9 | 5 | 639 | 70.0 | 5.74 | 488.15 | Heat shock-related 70 kDa protein 2 |
| **IPI:IPI00339269.1** | 9.02 | 10 | 5 | 643 | 71.0 | 6.14 | 486.05 | Heat shock 70 kDa protein 6 |
| **IPI:IPI00930688.1** | 34.59 | 10 | 10 | 451 | 50.1 | 5.06 | 477.18 | Tubulin alpha-1B chain |
| **IPI:IPI00013683.2** | 18.22 | 8 | 6 | 450 | 50.4 | 4.93 | 469.11 | Tubulin beta-3 chain |
| **IPI:IPI00220327.4** | 13.04 | 7 | 7 | 644 | 66.0 | 8.12 | 455.87 | Keratin, type II cytoskeletal 1 |
| **IPI:IPI00441473.3** | 22.92 | 11 | 10 | 637 | 72.6 | 6.29 | 435.00 | Protein arginine N-methyltransferase 5 |
| **IPI:IPI00332511.5** | 25.73 | 8 | 8 | 447 | 51.7 | 6.20 | 434.16 | Serine/threonine-protein phosphatase 2A 55 kDa regulatory subunit B alpha isoform |
| **IPI:IPI00011200.5** | 19.32 | 8 | 8 | 533 | 56.6 | 6.71 | 352.67 | D-3-phosphoglycerate dehydrogenase |
| **IPI:IPI00646779.2** | 10.07 | 4 | 3 | 447 | 50.1 | 4.89 | 272.71 | TUBB6 protein |
| **IPI:IPI01011853.1** | 12.03 | 4 | 3 | 374 | 41.9 | 4.91 | 246.39 | cDNA FLJ35358 fis, clone PUAEN2000497, highly similar to Tubulin beta-6 chain |
| **IPI:IPI00019359.4** | 8.03 | 4 | 4 | 623 | 62.0 | 5.24 | 226.26 | Keratin, type I cytoskeletal 9 |
| **IPI:IPI00410017.1** | 8.96 | 3 | 3 | 547 | 61.1 | 9.07 | 204.84 | Isoform 2 of Polyadenylate-binding protein 1 |
| **IPI:IPI01014861.1** | 7.64 | 3 | 3 | 615 | 65.2 | 9.13 | 200.09 | Heterogeneous nuclear ribonucleoprotein M isoform a variant (Fragment) |
| **IPI:IPI00021304.1** | 5.12 | 3 | 3 | 645 | 65.8 | 8.00 | 188.62 | Keratin, type II cytoskeletal 2 epidermal |
| **IPI:IPI01012502.2** | 8.29 | 3 | 3 | 567 | 62.2 | 9.01 | 179.54 | Uncharacterized protein |
| **IPI:IPI00027251.1** | 5.38 | 2 | 2 | 465 | 54.2 | 7.15 | 169.48 | Serine/threonine-protein kinase 38 |
| **IPI:IPI00291510.3** | 8.56 | 3 | 3 | 514 | 55.8 | 6.90 | 159.61 | Inosine-5'-monophosphate dehydrogenase 2 |
| **IPI:IPI00293665.9** | 3.90 | 2 | 2 | 564 | 60.0 | 8.00 | 140.51 | Keratin, type II cytoskeletal 6B |
| **IPI:IPI00893179.1** | 2.51 | 1 | 1 | 557 | 64.0 | 6.83 | 136.33 | X-ray repair complementing defective repair in Chinese hamster cells 6 |
| **IPI:IPI00964764.1** | 5.84 | 3 | 3 | 616 | 67.3 | 8.12 | 133.46 | cDNA FLJ55072, highly similar to Succinate dehydrogenase (ubiquinone) flavoprotein subunit, mitochondrial |
| **IPI:IPI00479145.3** | 6.75 | 3 | 3 | 400 | 44.1 | 5.14 | 119.56 | Keratin, type I cytoskeletal 19 |
| **IPI:IPI00980222.1** | 8.25 | 2 | 2 | 315 | 34.8 | 5.76 | 113.71 | Uncharacterized protein |
| **IPI:IPI01022477.1** | 5.96 | 1 | 1 | 151 | 16.6 | 7.81 | 108.33 | Uncharacterized protein |
| **IPI:IPI00879638.2** | 5.95 | 3 | 2 | 420 | 47.5 | 7.66 | 104.21 | cDNA FLJ58652, highly similar to Probable ATP-dependent RNA helicase DDX17 |
| **IPI:IPI00216773.6** | 6.06 | 3 | 2 | 396 | 45.1 | 6.10 | 103.57 | Uncharacterized protein |
| **IPI:IPI00982782.1** | 17.00 | 1 | 1 | 100 | 11.2 | 6.79 | 101.05 | Uncharacterized protein |
| **IPI:IPI00413691.2** | 9.14 | 1 | 1 | 197 | 21.9 | 9.55 | 89.90 | polypyrimidine tract-binding protein 1 isoform d |
| **IPI:IPI00985384.1** | 3.72 | 2 | 2 | 646 | 71.3 | 6.62 | 87.43 | ATP-dependent RNA helicase DDX3X isoform 3 |
| **IPI:IPI00471928.6** | 1.99 | 1 | 1 | 503 | 54.5 | 8.24 | 81.89 | ATP synthase subunit alpha |
| **IPI:IPI00020127.1** | 2.11 | 1 | 1 | 616 | 68.1 | 7.21 | 75.47 | Replication protein A 70 kDa DNA-binding subunit |
| **IPI:IPI01015265.1** | 6.35 | 2 | 2 | 378 | 43.3 | 6.48 | 68.42 | cDNA FLJ51908, highly similar to Dolichyl-diphosphooligosaccharide--proteinglycosyltransferase 67 kDa subunit |
| **IPI:IPI00916188.1** | 7.41 | 1 | 1 | 297 | 32.4 | 5.36 | 67.13 | 32 kDa protein |
| **IPI:IPI01021839.1** | 9.77 | 1 | 1 | 133 | 13.9 | 5.82 | 65.54 | 14 kDa protein |
| **IPI:IPI00910786.2** | 8.44 | 1 | 1 | 237 | 26.0 | 9.22 | 65.41 | Uncharacterized protein |
| **IPI:IPI01022121.1** | 2.88 | 1 | 1 | 416 | 44.8 | 6.54 | 58.03 | Uncharacterized protein |
| **IPI:IPI00909657.1** | 2.94 | 1 | 1 | 477 | 54.1 | 7.80 | 53.59 | cDNA FLJ50378, highly similar to Phenylalanyl-tRNAsynthetase alpha chain |
| **IPI:IPI01014133.1** | 2.87 | 1 | 1 | 418 | 46.3 | 5.44 | 52.82 | cDNA FLJ32936 fis, clone TESTI2007533, highly similar to RuvB-like 2 |
| **IPI:IPI00922068.1** | 2.84 | 1 | 1 | 493 | 54.7 | 7.52 | 51.61 | Isoform 5 of Insulin-like growth factor 2 mRNA-binding protein 2 |
| **IPI:IPI00981988.1** | 33.33 | 1 | 1 | 51 | 5.8 | 5.31 | 48.06 | Uncharacterized protein |
| **IPI:IPI00478966.3** | 5.32 | 1 | 1 | 188 | 21.5 | 8.03 | 47.23 | Uncharacterized protein |
| **IPI:IPI00978175.1** | 1.99 | 1 | 1 | 603 | 68.1 | 8.90 | 46.42 | Uncharacterized protein |

**Full-length gels and blots of main figures**

**
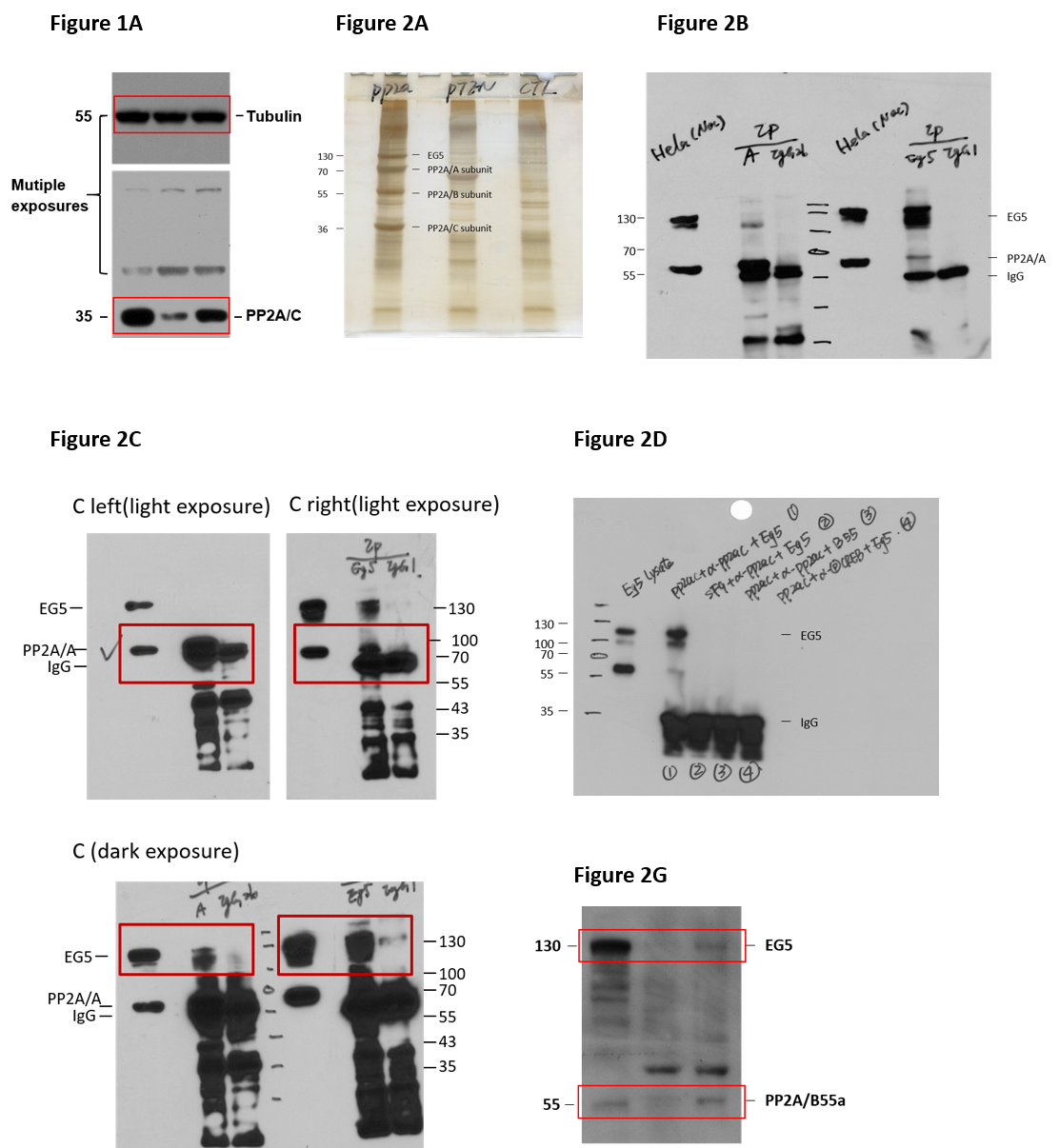
**

**
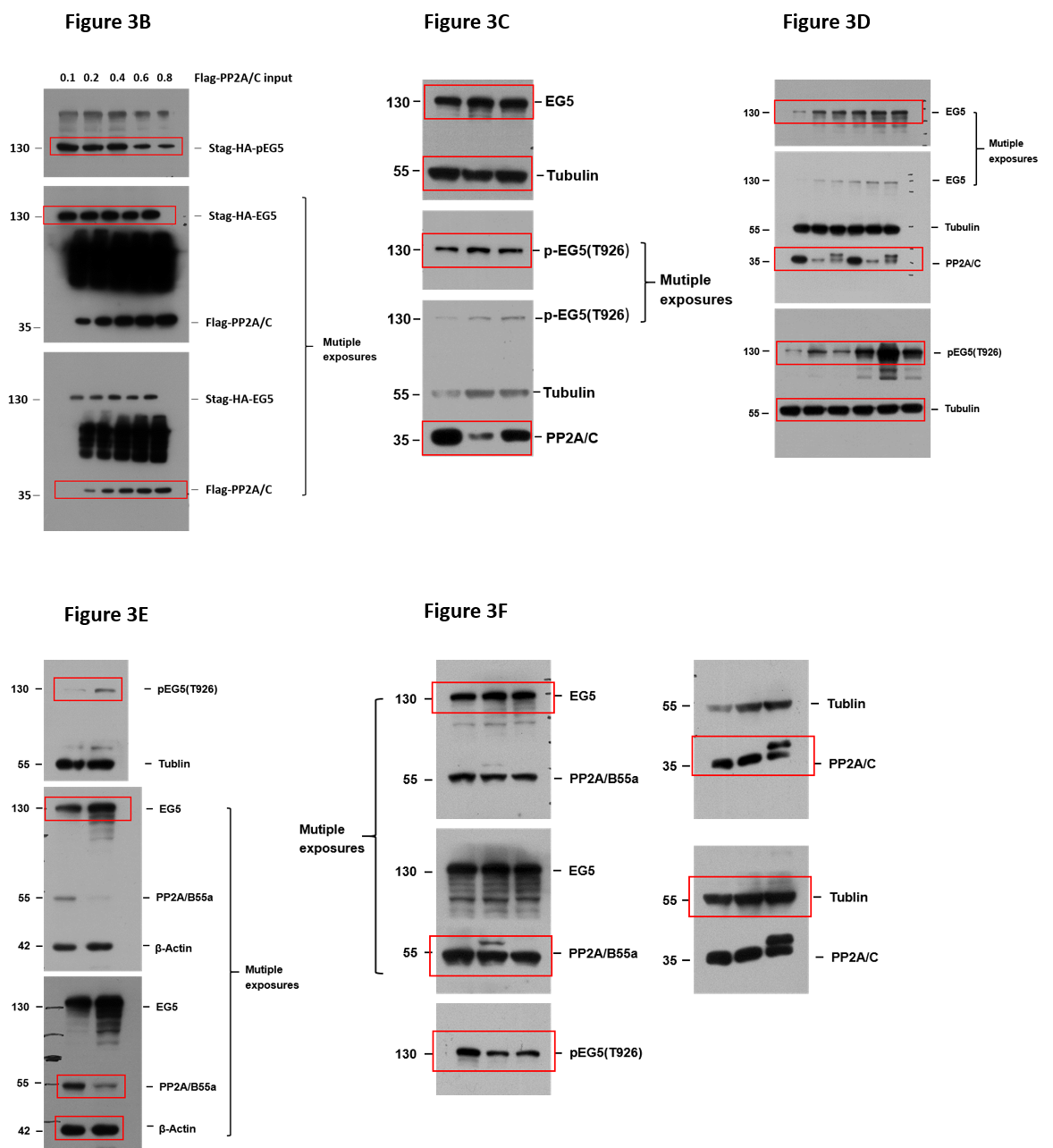

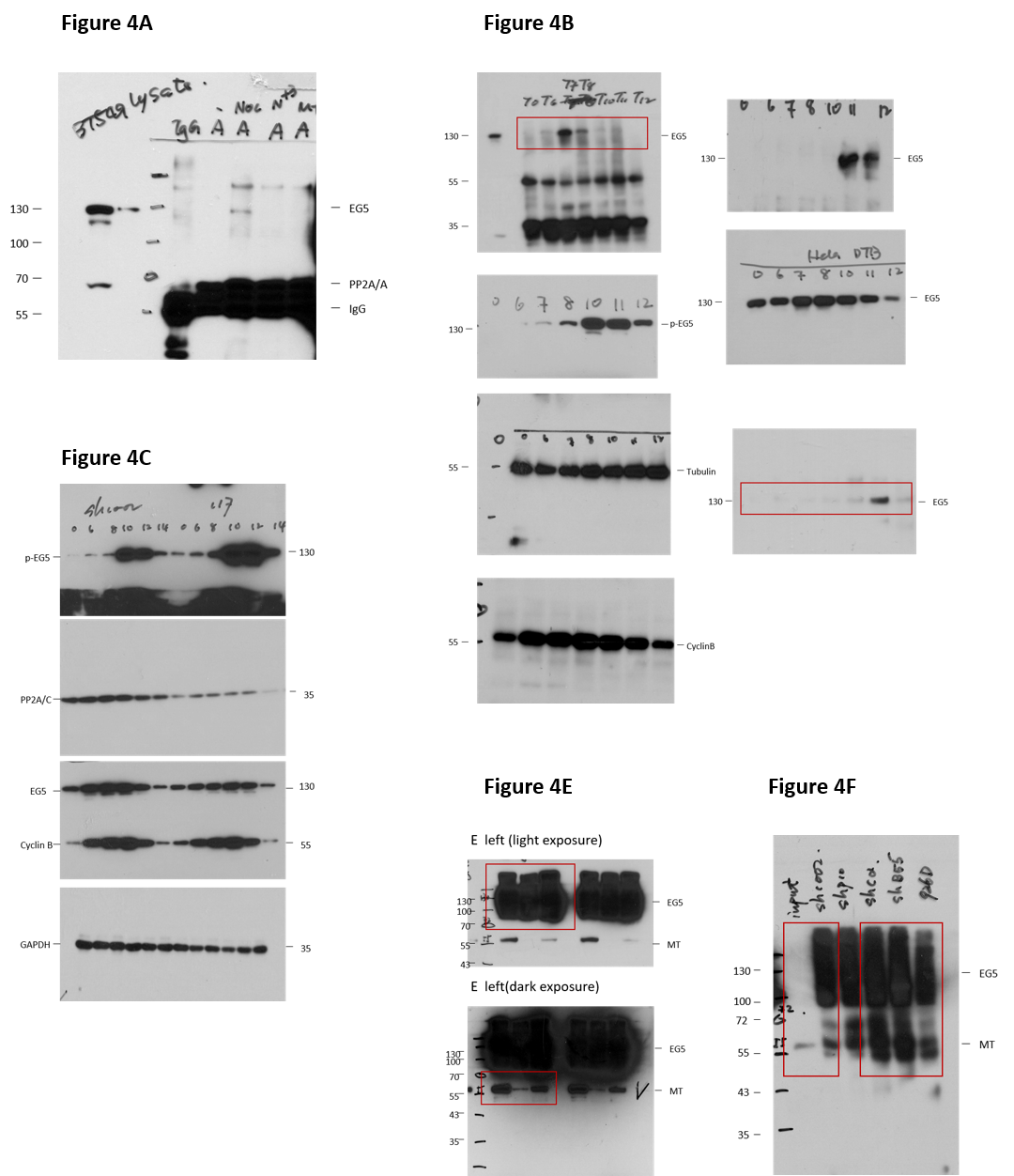
**
